# *In vitro* culture of olfactory epithelial cells from *Megalobrama amblycephala* and their response to amino acid mixtures and prostaglandin F_2α_

**DOI:** 10.3389/fcell.2025.1587151

**Published:** 2025-09-11

**Authors:** Suhua Guan, Xin Huang, Dongmei Zhu, Zexia Gao, Han Liu

**Affiliations:** ^1^ Key Lab of Agricultural Animal Genetics, Breeding and Reproduction of Ministry of Education / Key Laboratory of Freshwater Animal Breeding, Ministry of Agriculture and Rural Affairs, College of Fisheries, Huazhong Agricultural University, Wuhan, China; ^2^ Hubei Hongshan Laboratory, Engineering Research Center of Green Development for Conventional Aquatic Biological Industry in the Yangtze River Economic Belt, Ministry of Education, Wuhan, China

**Keywords:** *Megalobrama amblycephala*, olfactory epithelium cells, *in vitro* culture, collagenase dissociation, olfactory receptors (ORs), prostaglandin F2α (PGF2α)

## Abstract

Olfaction is essential for the survival and reproduction of fish, as it facilitates foraging, food localization, mate selection, and breeding. The *in vitro* cultured olfactory epithelial cells will provide an important resource for research on how fish use olfaction to detect odor molecules in their environment. In this study, olfactory epithelial cells from *Megalobrama amblycephala* were cultured *in vitro* to investigate their responses to various odors, amino acids, and prostaglandin F_2α_ (PGF_2α_). Initially, the olfactory epithelial cells were cultured *in vitro* using the explant method and collagenase digestion technique. Based on observations of *in vitro* growth characteristics, collagenase digestion demonstrated superior growth stability and morphological features of ciliated neurons. The presence of olfactory neurospheres was identified through scanning electron microscopy (SEM). Immunofluorescence analysis revealed that most of the cells cultured were labeled with NEUN antibody. Additionally, the expression of olfactory receptors (*ORs*) was detected in the *in vitro* cultured olfactory epithelial cells using fluorescence *in situ* hybridization (FISH) and reverse transcription PCR (RT-PCR). Stimulation with amino acids mixture and PGF_2α_ significantly increased the number of olfactory epithelial cells labeled with pERK. RNA-seq analysis revealed that 1,276 differentially expressed genes (DEGs) were identified following PGF_2α_ stimulation, with pathways related to olfaction and reproduction being significantly enriched. Collectively, this study successfully established an *in vitro* model of the olfactory epithelium cells in *M. amblycephala* and preliminarily investigated its response to odorant molecules, providing a valuable platform for research on fish olfactory function.

## 1 Introduction

Olfaction plays a crucial role in the sensory system of vertebrates, encompassing a wide range of intricate functions (Hughes et al., 2018). In fish behavior, it is essential for foraging, mating, predator evasion, and migratory activities. The detection of odors relies on the expression of olfactory receptors (*ORs*) in olfactory sensory neurons (OSNs) (Bazáes et al., 2013; Hughes et al., 2018). OSNs utilize axons to perceive odor molecules and transmit impulses to the brain, thereby integrating olfactory functions (Firestein, 2001; Soelter et al., 2020).

OSNs express *ORs* that have evolved to detect odorants relevant to behavior. Throughout vertebrate evolution, *ORs* have undergone significant expansion (Policarpo et al., 2024). This increasing diversity is exemplified by the growth from an estimated 159 *ORs* in zebrafish to 2,000 *ORs* in African elephants (Niimura et al., 2014). Importantly, each functional *ORs* selectively identifies a unique odorant molecule (Braubach et al., 2012), while individual OSNs persistently express a singular *OR* (Andrew et al., 1994). Furthermore, OSNs possess a unique regenerative capability that distinguishes them from other types of neural cells in mammals (Suzuki and Takeda, 1993), playing a vital role in the sense of smell. However, culturing OSNs *in vitro* presents significant challenges. Some studies suggest that the viability and functional integrity of mature OSNs may be adversely affected by enzyme digestion during their cultivation (Schwarzenbacher et al., 2005; Lacroix et al., 2008). Previous studies have established primary culture systems using cells derived from embryonic, neonatal or adult mice for cultivation of OSNs (Micholt et al., 2012). Cultured olfactory stem cells have been successfully obtained from cloned mice (Peterson et al., 2019) and human neurospheres (Murrell et al., 2005). However, the differentiation of these cells into mature OSNs has presented a significant challenge. The COVID-19 pandemic has sparked significant interest in the investigation and cultivation of OSNs (Hao et al., 2020; Wu et al., 2023). Researchers have developed chemically and mechanically-based models to explore the crucial functions of OSNs in mammals (Ren et al., 2021). Considering that viruses can inflict damage on the olfactory system (Hou et al., 2020; Ahn et al., 2021), extensive research efforts have been dedicated to investigating their impact, leading to enhanced comprehension. For example, found that the virus attaches to motile cilia via the ACE2 receptor (Wu et al., 2023). Recent studies have challenged the conventional belief that OSNs cannot survive outside an organism, thereby overturning this long-standing notion (Gao et al., 2021; Ren et al., 2021). Simultaneously, we acknowledge the imperative of cultivating OSNs for studying olfactory function.

Fish, which constitute one of the largest categories in vertebrates, account for approximately half of all known species. They primarily rely on detecting water-soluble odorant molecules such as amino acids, prostaglandin F_2α_ (PGF_2α_), and bile acids to ensure their survival in aquatic environments (Cong et al., 2019). Moreover, there is an increasing recognition that the olfactory capabilities of fish are intricately linked to their dietary habits and reproduction (Liu et al., 2021). However, the ability to culture fish OSNs remains relatively limited. As a representative herbivorous fish species, the *Megalobrama amblycephala* possesses a considerable number of *ORs* (Liu et al., 2021). In this study, we focused on *M. amblycephala*, a fish species of economic importance. We employed a combination of Collagenase I + IV and trypsin to isolate olfactory epithelium from *M. amblycephala.* This method effectively reduces the damage to neuronal cilia. Through the synergistic application of these three enzymes, we achieved targeted and thorough digestion of distinct olfactory epithelium regions, enhancing its applicability. This work represents a preliminary exploration of culturing olfactory epithelial cells in aquatic organisms.

## 2 Materials and methods

### 2.1 Sample collection and maintenance

Cells isolated from five *M. amblycephala*, both females and males, were used simultaneously. Twelve-month-old *M. amblycephala*, with an average body weight of 300 ± 50 g, were sourced from the Ezhou Aquaculture Base in Wuhan and subsequently housed in the recirculating aquaculture system at the Huazhong Agricultural University Aquaculture Base. Individuals exhibiting excellent health underwent a preventive treatment involving a 20 min immersion in water containing potassium permanganate at a concentration of 0.5 ppm before being introduced into the recirculatory system. Throughout the maintained period, dissolved oxygen levels were maintained at 8 ± 3 mg/L, and the water temperature was regulated to be within the range of 24 °C ± 4 °C.

### 2.2 Explant isolation of olfactory epithelium

Prior to performing aseptic procedures, *M. amblycephala* specimens were euthanized using anesthesia and exsanguinated. The carcasses were then disinfected with alcohol to prevent bacterial and fungal contamination. On a sterile workbench, the olfactory epithelium tissues were meticulously excised using sterilized tweezers and forceps before being transferred to a sterile culture dish. Any remaining mucus tissue was removed through two consecutive rinses with PBS.

The olfactory epithelium tissues were incubated in Antibiotic incubation medium (Table 1) for 2 h, followed by meticulous dissection using sterilized knives and scissors on a sterile Petri dish. Approximately thirty tissue fragments, each measuring 1–2 mm^3^, were evenly distributed in a T25 culture flask and then the growth medium was added. The flask was positioned vertically for 2 h, then tilted horizontally for 30 min to facilitate tissue adhesion. Subsequently, 3 mL of the growth medium was introduced. Cultures were maintained at 28 °C, with the growth medium refreshed every 3 days. Daily assessment of fragment adherence, dispersion, and proliferation was facilitated using an inverted phase contrast microscope.

**TABLE 1:** Medium composition.

Culture medium	Composition	
	Content	Volume
Antibiotic incubation medium (AIM)	pen strep (Gibco, catalog number: 15070063)	2 mL
	amphotericin B (1 mg/mL) (CAS:1,397–89–3)	1 mL
	gentamicin sulfate (2 mg/mL) (CAS:1,405–41–0)	2.5 mL
	DMEM/F12 (Gibco, catalog number: 11320033)	up to 50 mL
Collagenase Ⅰ + Ⅳ	collagenase Ⅰ (Biosharp, catalog number: BS163)	100 mg
	collagenase Ⅳ (Biosharp, catalog number: BS165)	100 mg
	PBS (Gibco, catalog number: 10010023)	up to 100 mL
Collagenase digestion solution	pen strep	2 mL
	amphotericin B	1 mL
	gentamicin sulfate	2.5 mL
	collagenase Ⅰ + Ⅳ	2 mL
	trypsin-EDTA (0.25%) (Gibco, catalog number: 25200056)	4 mL
	DMEM/F12	up to 50 mL
Neutralization medium	pen strep	2 mL
	amphotericin B	0.5 mL
	gentamicin sulfate	2.5 mL
	FBS (Fetal bovine serum) (Cell-Box, catalog number: AUS-02S-02)	10 mL
	DMEM/F12	up to 50 mL
Growth medium	pen strep	2 mL
	amphotericin B	0.5 mL
	gentamicin sulfate	2.5 mL
	B-27 Supplement (Gibco, catalog number: 17504044)	1 mL
	N-2 Supplement (Gibco, catalog number: 17502048)	0.5 mL
	HEPES (Gibco, catalog number: 15630106)	0.5 mL
	FBS	5 mL
	DMEM/F12	up to 50 mL
Neuronal culture medium	pen strep	2 mL
	B-27	1 mL
	N-2	0.5 mL
	HEPES	0.5 mL
	Neurobasal™ Plus Medium (Gibco, catalog number: A35829-01)	up to 50 mL

### 2.3 Collagenase isolation of olfactory epithelium

**FIGURE 1:**
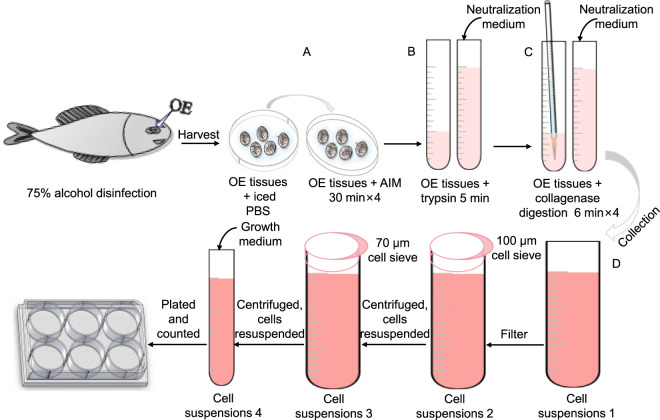
Schematic representation of the collagenase isolation protocol for olfactory epithelium (OE). **(A)** OE tissues were rinsed with PBS and subsequently immersed in AIM solution for thorough sterilization. **(B)** Digestion with trypsin-EDTA (0.25%) was performed to remove the surrounding mucus. **(C)** Collagenase digestion solution was applied to the OE tissues, followed by gentle pipetting with a 5 mL transfer pipette to enhance dissociation, with this process being repeated four times. **(D)** The cell suspension was collected and placed on ice, followed by filtration and subsequent inoculation.

The olfactory epithelium tissue was incubated in AIM within a 50 mL centrifuge tube for 2 h to ensure complete saturation. Subsequently, the surrounding mucus was eliminated by treating the tissue with trypsin-EDTA (0.25%) for 5 min. After trypsinization, a neutralization medium was added to halt enzymatic activity, and then the supernatant was discarded. The tissue was then gently pipetted in the presence of collagenase working solution to facilitate further digestion. This enzymatic treatment lasted for 6 min, following which neutralization medium was introduced and the supernatant containing dissociated cells was collected while retaining the tissue for subsequent processing. The digestion was repeated four times to ensure thorough tissue disintegration thoroughly, with additional digestion performed as necessary until complete digestion confirmation of digestion. All supernatants were filtered through a 100 μm cell strainer to remove undigested materials and subsequently centrifuged for cell isolation. The resulting cells were washed in neutralization medium within a 15 mL centrifuge tube and passed through a 70 μm cell strainer. Following a second centrifugation step, the cells were resuspended in growth medium, and their concentration was determined using an automated cell counter. Finally, the cells were plated at a density of 1 × 10^6^ cells/mL in sterile six-well plates and incubated in a 28 °C incubator with 5% CO_2_ (Figure 1). After 24 h, the initial growth medium was replaced with neuronal cell culture medium, repeating this replacement process every 3 days. Table 1 presents the formulation of all culture media.

### 2.4 Morphology and vitality assay of olfactory epithelial cells

The morphology of olfactory epithelial cells during the growth phase was monitored using inverted phase contrast microscopy, with initial observations commencing on the first day of growth and continuing twice daily. For detailed morphology assessment, olfactory epithelial cells were analyzed using Scanning Electron Microscopy (SEM). Samples designated for SEM analysis were fixed overnight at 4 °C in 2.5% glutaraldehyde (EM Grade) and subsequently underwent a secondary fixation in 2% osmium tetroxide (OsO_4_) for 2 h at room temperature. Following fixation, samples were progressively dehydrated through a series of ethanol immersions at concentrations of 30%, 50%, 70%, 95%, and finally 100%. They were then critically point dried using hexamethyldisilazane (HMDS). The dry specimens were metal-coated and examined with a Field Emission Scanning Electron Microscope (FESEM).

The proliferation activity of olfactory epithelial cells was assessed using the Cell Counting Kit-8 (CCK-8) assay. Cells were seeded at a concentration of 1 × 10^6^ cells/mL in a 96-well plate and divided into seven groups with ten replicate wells per group. Proliferation was monitored over 7 days, with daily medium refreshment. At specified time points, each well was incubated with 10 µL of CCK-8 solution for 4 h, followed by absorbance measurement at 450 nm using a microplate reader (Envision).

### 2.5 Neuronal nuclei (NEUN) detection *in Vitro* cultured olfactory epithelial cells

On the sixth day of culturing olfactory epithelial cells on adhesive glass coverslips with a diameter of 8.5 mm, the culture medium was discarded, and cells were washed with PBS to remove residual medium and impurities. The olfactory epithelial cells were fixed in 4% paraformaldehyde for 15 min, followed by another wash with PBS. Permeabilization was achieved by treating the cells with 0.1% Triton X-100 for 20 min. Subsequently, the cells were blocked for 1 h in PBS containing 0.01% Triton X-100, enriched with 2.5% goat serum, 2.5% donkey serum, and 20 mM glycine. An overnight incubation at 4 °C followed using a rabbit NeuN antibody (GeneTex, catalog number: 43,579) diluted to a concentration of 1:1,000 in the blocking solution. The following day, cells were acclimatized to room temperature for 30 min, washed with PBS, and then incubated in darkness for 1 h with Alexa Fluor 488-conjugated secondary antibody (Donkey anti-rabbit at 1:500 dilutions, AntGene, catalog number: 1650451008). After washing, cells were stained with DAPI for 5 min to visualize nuclei and rinsed to remove excess stain. Finally, the cell slips were treated with antifade mounting medium and examined using a laser confocal microscope.

### 2.6 Detection of olfactory receptors (*ORs*)

To determine whether olfactory epithelial cells *in vitro* express *ORs*, fluorescence *in situ* hybridization (FISH) and RT-PCR were utilized for detection. The entire coding sequence of the *OR-β11* from *M. amblycephala* was amplified from cDNA, cloned into the pcDNA3.1 (+) vector, and sequenced. Probes for *OR-β11* were generated by amplifying the gene fragment from the cloning vector using primers listed in Supplementary Table S1. An antisense cRNA probe targeting OR-β11 was synthesized *in vitro* using T7 RNA polymerase and enhanced with a DIG RNA labeling mixture. Cell slides were fixed with 4% PFA for 15 min. After washing with PBST, they were treated with 0.1% Triton X-100 for 10 min. Following another PBST wash, slides were pre-hybridized at 65 °C for 1 h in hybridization buffer (50% formamide, 5×SSC, 0.05 mg/mL heparin, 0.5 mg/mL tRNA, 0.1% Tween-20, 0.01 M citric acid, DEPC-treated). The *OR-β11* probe (5 ng/μL) was then added and hybridized at 65 °C for 12–16 h. After hybridization, slides were washed in Wash Solution I (25% formamide, 1×SSC, 0.1% Tween-20) for 2 × 30 min, in Wash Solution II (1×SSC, 0.1% Tween-20) for 2 × 15 min, and in Wash Solution III (0.2×SSC, 0.1% Tween-20) for 2 × 30 min. Slides were cooled to room temperature and incubated with Anti-DIG POD Fab fragments (1:500, Roche) at 4 °C for 16–18 h, followed by PBST washes for 3 × 5 min. Then the slides were processed using the Tyramide Signal Amplification (TSA) Cy3 Kit (Akoya Biosciences) according to the manufacturer’s instructions. Following a 10 min incubation with Cy3-conjugated tyramide (diluted 1:50 in amplification buffer) at room temperature, the slides were subjected to 3 × 5 min washes in PBST. Finally, slides were stained with DAPI (5 μg/mL) for 10 min and mounted with an anti-fade mounting solution. After examination under a confocal microscope (Nikon), all cell slides were scanned using a high-resolution slide scanning system (3DHISTECH Ltd.).

RNA was extracted from olfactory epithelial cells cultured *in vitro* for 6 days using trizol (TaKaRa, catalog number: 9,108). Subsequently, the RNA was reverse transcribed into cDNA using the Prime Script RT Reagent Kit with gDNA Eraser (TaKaRa, catalog number: RR047A) as the template for RT-PCR. The reaction volume was 10 μL, containing 5 μL of 2 × Rapid Taq Master Mix (Vazyme, catalog number: P222-01), 0.2 mM primer, and cDNA template. The cycling program was as follows: initial denaturation at 95 °C for 3 min, denaturation at 95 °C for 15 s, annealing at 58 °C for 15 s, extension at 72 °C for 3 s, repeated for 35 cycles. Finally, the RT-PCR products were analyzed by 1% agarose gel electrophoresis.

### 2.7 Detection of pERK *in vitro* cultured olfactory epithelial cells after exposure to odorant molecules

The odorants were sourced from Sigma-Aldrich with a purity level of 97%. The amino acids mixture, used as food odor, was based on the composition of amino acids in water grass, as shown in Supplementary Table S1. Each experimental session began with a freshly prepared solution containing the mixture. PGF_2α_, as a sex pheromone, was dissolved in DMSO to achieve a concentration of 1 × 10^−1^ M and stored at −80 °C for future use. Fresh neural cell culture medium was replaced for the six-day-old olfactory epithelial cells 12 h prior to initiating the odor exposure experiment. Subsequently, an appropriate amount of the amino acid mixture solution and PGF_2α_ were added to reach a final concentration of 1 × 10^−5^ M. The olfactory epithelial cells were then incubated at 28 °C in a culture chamber. After a 10 min exposure period, ERK phosphorylation was confirmed using rabbit anti-pERK1/2 monoclonal antibody (1:500, Cell Signaling Technology, catalog number: 4,370). Three biological replicates were set up for each experiment. The experimental procedures were as described in section 2.5. Subsequent quantification was performed using ImageJ software (v1.53t) under standardized parameters. For nuclear enumeration, DAPI-stained nuclei were counted. pERK^+^ cells were identified as those exceeding the intensity threshold (mean + 2SD of controls). The positivity rate was calculated using the formula: % pERK^+^ = (pERK^+^ cells/total nuclei) × 100. Each group included a minimum of three replicates.

### 2.8 RNA-seq and analysis

After a 2 h exposure to a solution containing amino acid mixture and PGF_2α_ at final concentration of 1 × 10^−5^ M, the olfactory epithelial cells were collected for total RNA extraction and subjected to quality evaluation. High-quality total RNA was utilized for constructing an RNA-seq library. The RNA was purified, reverse transcribed, and libraries were constructed following the manufacturer’s instructions (Illumina, San Diego, CA). Briefly, the transcriptome library for olfactory epithelial cells was prepared using the Illumina Stranded mRNA Prep Kit with 1 μg of total RNA. Subsequently, messenger RNA was isolated through a poly A selection method employing oligo (dT) beads and fragmented using a fragmentation buffer. Next, double-stranded cDNA synthesis was performed utilizing the Super Script Double-Stranded cDNA Synthesis Kit (Invitrogen, CA) with Illumina random hexamer primers. The synthesized cDNA underwent end repair, phosphorylation, and “A” base addition according to Illumina’s library construction protocol. Size selection of the resulting cDNA libraries targeted fragments of 300 bp on 2% Low Range Ultra Agarose gel followed by PCR amplification consisting of 15 cycles using Phusion DNA Polymerase from NEB. After quantification with the Qubit 4.0 Fluorometer, paired-end RNA-seq sequencing library was sequenced using the Nova Seq 6,000 sequencer. The raw data underwent filtration to remove sequencing reads containing adapters, poly-N sequences, and low-quality sequences (Q < 20). The remaining reads were defined as “clean reads” and utilized for subsequent analysis. Subsequently, the clean reads were mapped to the reference genome of *M. amblycephala* (PRJNA343584) using HISAT. Following this, the RSEM program was employed to quantify gene expression, with gene expression levels presented in the form of transcripts per million (TPM).

To identify differentially expressed genes (DEGs) between two groups, we utilized the transcripts per million reads (TPM) method to quantify the expression level of each transcript. Gene abundances were quantified using RSEM. Subsequently, differential expression analysis was conducted using DESeq2. Genes with |log2FC|≥1 and FDR <0.05 (DESeq2) were considered as significantly differentially expressed genes. Furthermore, functional enrichment analysis for Gene Ontology (GO) and Kyoto Encyclopedia of Genes and Genomes (KEGG) was conducted to identify significantly enriched DEGs in GO terms and metabolic pathways, respectively. The Bonferroni-corrected *p*-value of 0.05 was used compared to the whole-transcriptome background. Goatools and Python scipy were employed for conducting the GO functional enrichment and KEGG pathway analysis, respectively.

### 2.9 Quantitative real-time PCR (qPCR)

Real-time quantitative PCR (qPCR) was performed using an ABI real-time qPCR system (Foster City, CA, United States of America) with a 20 μL reaction volume containing 10 μL SYBR Green PCR Master Mix (TaKaRa, code No. RR820A), 0.4 mM primers and the cDNA template. The cycling program consisted of an initial denaturation for 5 min at 95 °C, followed by 40 cycles of 95 °C for 20 s and 60 °C for 25 s *β-actin* was utilized as a reference gene, using its expression level as a standard to measure the relative expression levels of the target gene. Subsequently, a comparison of the relative expression levels among samples was conducted, with DMSO stimulation serving as the control group. The dataset was systematically organized and analyzed utilizing Microsoft Excel 2010, employing the 2^−ΔΔCt^ method (Schmittgen and Livak, 2008) for the quantitative analysis of the data. The primers utilized in this study can be found in Supplementary Table S2.

### 2.10 Statistical analyses

The results were expressed as the mean ± standard deviation (mean ± SD) across three independent experimental replicates. After normalization, the data underwent one-way analysis of variance (One-Way ANOVA) using SPSS 19.0 software. Subsequently, multiple comparisons were performed using the Scheffé method. Statistical significance was determined at *P* < 0.05 and *P* < 0.01, indicating significant and highly significant differences, respectively.

## 3 Results

### 3.1 Cells derived from explants exhibit distinct *in vitro* growth characteristics

In the explant culture system, we observed two distinct phenomena. On day 7 of culturing, a subset of the explants exhibited simultaneous radial outgrowth of both neuronal-like and paving-stone like cells, albeit with slow cell proliferation. By day 15, the number of bipolar neuron-like cells ceased to increase, while the number of dead cells began to rise (Supplementary Figure S1A). In contrast, another set of explants, there was a profusion of paving-stone like cells appearing by day 3. These cells started detaching by day 5, and notably, there was no migration of neuronal-like cells during the subsequent culture period (Supplementary Figure S1B).

### 3.2 Olfactory epithelial cells isolated using collagenase exhibit stable growth *in vitro*

**FIGURE 2:**
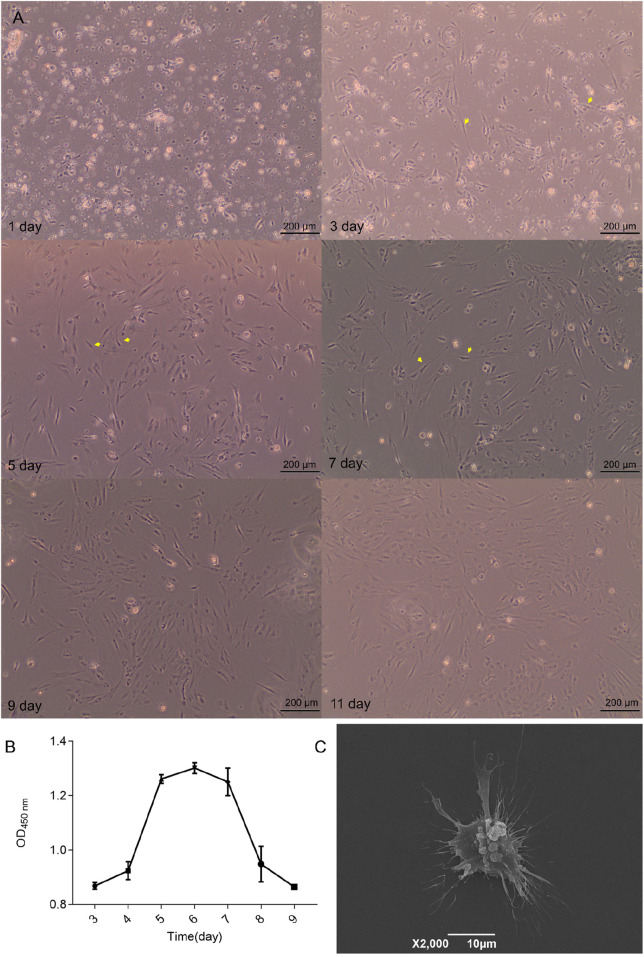
Characteristics of olfactory epithelial cells. **(A)** The growth conditions of cells observed via phase-contrast microscopy. **(B)** Cell viability was evaluated systematically over seven consecutive days utilizing the CCK-8 assay. **(C)** Examination of cell morphology utilizing SEM.

Olfactory epithelium tissues from five *M. amblycephala* specimens were completely digested using a collagenase digestion solution. The resulting cells were resuspended in growth medium and seeded into a six-well culture dish at a concentration of 1 × 10^6^ cells/mL. After approximately 24 h of seeding, most majority olfactory epithelial cells adhered to the surface of Petri dishes (Figure 2A). By the third day, cell proliferation occurred with some displaying typical bipolar neuronal morphology. This bipolar morphology exhibited a significant increase by the fifth day and persisted for approximately 72 h. However, on day nine, there was a decline in the abundance of bipolar neurons coinciding with an increase in round cells. Concurrently, cell viability as determined by the CCK-8 assay demonstrated a substantial decrease on day eight (Figure 2B). The exponential phase of cell growth occurred on day five, reaching peak viability on day six. Notably, by day eight, there was a significant reduction in viability which corroborated morphological assessments of the cells. SEM conducted on day six revealed olfactory neurospheres featuring filamentous extensions resembling cilia encircling them (Figure 2C).

### 3.3 Olfactory epithelial cells were labeled with the NEUN antibody and expressed *ORs*

**FIGURE 3:**
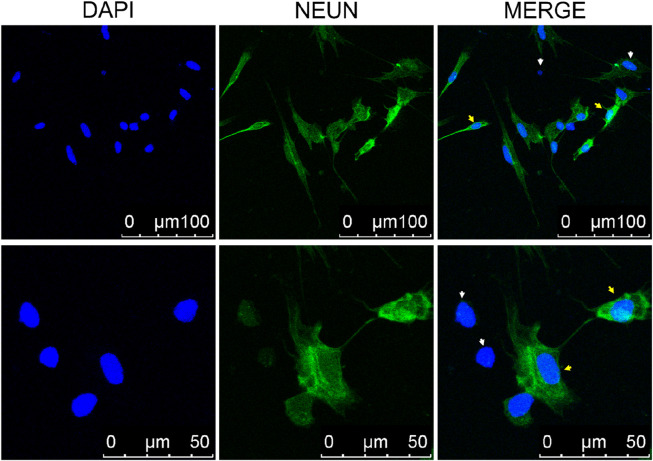
Laser Scanning Confocal Microscope of immunofluorescence staining of neuronal cells. The neurons were labeled with anti-NEUN antibodies, which appear green in the nucleus (indicated by yellow arrows). Cells nuclear were stained with DAPI (blue). The white arrow indicates non-neuronal cells.

To detect neurons within the olfactory epithelial cells, labeling was performed using the NEUN antibody, a specific marker for neuronal nuclei. The olfactory epithelial in fish primarily consists of neurons, supporting cells, and basal cells, all crucial for the development and signal transduction processes within OSNs. It was hypothesized that these cellular components would be present in the cultured olfactory epithelial cells. Therefore, NeuN antibodies were utilized to selectively label cells. The results demonstrated that the nuclei of majority of cells were bright green fluorescently labeled, suggesting a significant presence of neurons in the cultured olfactory epithelium cells (Figure 3). The unlabeled cells were non-neuronal, such as basal cells.

Following the identification of neurons within the cultured olfactory epithelial cells, the expression of *ORs* was assessed. Initially, FISH revealed the presence of *OR-β11* (*OR114-1*) (Supplementary Figure S2A). Subsequently, RT-PCR detected the expression of multiple *ORs*, including *OR-β1*, *OR-β2*, *OR-β9*, *OR-β10*, *OR-β11*, *OR-β12*, *OR-β7*, *OR-β8*, and *OR-β15* (Supplementary Figure S2B).

### 3.4 Olfactory epithelial cells are activated by amino acid mixture and PGF_2α_

**FIGURE 4:**
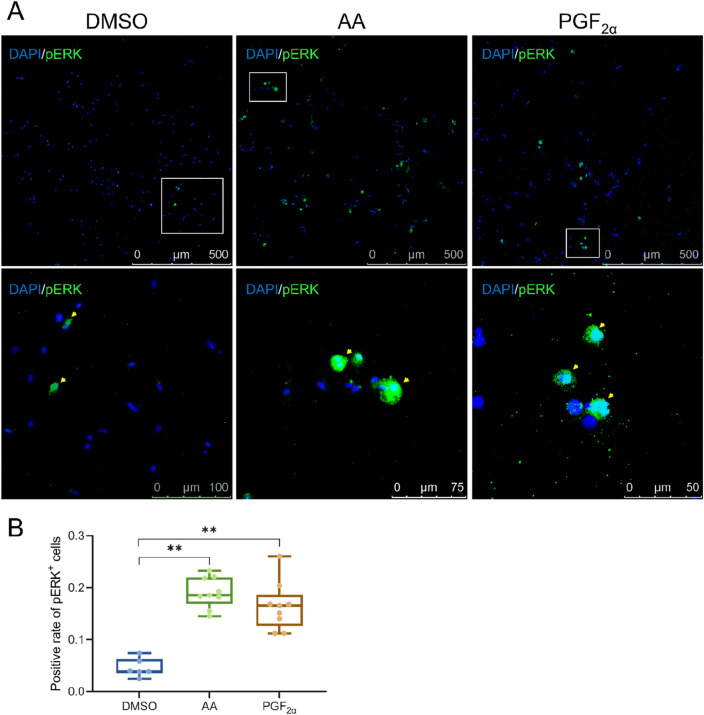
A small number of cells activated by the amino acid mixture and PGF_2α_ were present among the olfactory epithelium cells. **(A)** Antibody staining of pERK in olfactory epithelium cells exposed to DMSO, AA (amino acid mixture), and PGF_2α_, respectively. **(B)** Quantification of activated olfactory epithelium cells. Odors with statistical significance are marked with * (*P* < 0.05) and highly significant by ** (*P* < 0.01).

To investigate the potential activation of olfactory epithelium cells by amino acids mixture and PGF_2α_, phosphorylated ERK (pERK) was utilized as a neuronal activation marker (Cui et al., 2025; Masuda et al., 2024; Wakisaka et al., 2017). Olfactory epithelium cells were exposed to odor molecules in the culture medium and subsequently incubated in a 28 °C 5% CO_2_ incubator for 10 min. Each odor molecule was tested with three biological replicates, while DMSO served as the control odor. The results revealed that the amino acid mixture produced the highest number of labeled cells, approximately 20%, while PGF_2α_ elicited a moderate response, with the proportion of labeled cells remaining below 20%. Therefore, exposure to the amino acid mixture and PGF_2α_ significantly increased the number of olfactory epithelium cells labeled with pERK (*P* < 0.01), indicating that the cultured olfactory epithelium cells possess olfactory recognition function when activated by these specific stimuli (Figure 4).

### 3.5 Distinct molecular characteristics of the cultured olfactory epithelial cells exposed to PGF_2α_

**FIGURE 5:**
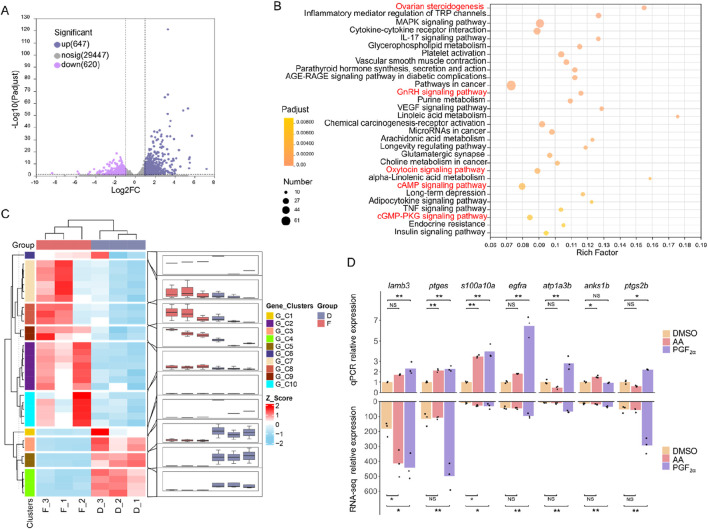
Transcriptome analysis of olfactory epithelium cells after exposure to PGF_2α_. **(A)** Log2-fold changes between DEGs after exposure to PGF_2α_ and DMSO, plotted based on their log10 FDR. **(B)** KEGG pathway enrichment of differentially expressed genes between PGF_2α_ and DMSO. Red shading indicates significant enrichment in key pathways associated with reproduction processes and olfactory signal transduction. **(C)** Heatmap depicting expression profiles of all 33 differentially expressed genes across three reproduction-associated pathways, clustered according to expression patterns. **(D)** The qPCR analysis quantified the expression of 7 DEGs. Data were normalized to *β-actin* as reference and presented as fold changes (mean ± SE) between the DMSO and amino acid mixture (AA) or PGF_2α_. Statistically significant differences are indicated by * (*P* < 0.05) and highly significant by ** (*P* < 0.01).

Following exposure to odor molecules, total RNA from cultured olfactory epithelium cells was extracted in three independent biological replicates for each odor (a total of 9 samples, n = 3). Each sample yielded an average of 6.09 GB clean data. These high-quality reads were successfully aligned to the genome of *M. amblycephala* with a mapping success rate surpassing 95%. PCA analysis (Supplementary Figure S3A) confirmed acceptable reproducibility within each treatment group and highlights discernible differences, particularly in the PGF_2α_ group. The distribution of gene expression levels in each sample and the degree of data dispersion were analyzed (Supplementary Figure S3B). The results demonstrated that the distribution of gene expression levels in the nine samples exhibited a reasonable pattern. Transcriptome analysis revealed that exposure to the amino acid mixture is with only 25 DEGs identified (Supplementary Figure S3C). In contrast, exposure to PGF_2α_ significantly differs from DMSO, resulting in a total of 1,267 DEGs (*P* < 0.05), including 647 upregulated and 620 downregulated genes (Figure 5A). KEGG pathway enrichment analysis revealed that 1,267 differentially expressed genes were significantly enriched in 59 pathways (*P* < 0.05), with the top 30 pathways shown (Figure 5B). Among these pathways, ovarian steroidogenesis, GnRH signaling pathway, and oxytocin signaling pathway are closely associated with reproduction. Ovarian steroidogenesis involves 20 DEGs, the GnRH signaling pathway involves 21 DEGs, and the oxytocin signaling pathway involves 27 DEGs, with a total of 33 non-redundant DEGs across these three reproductive pathways (Figure 5C). Specifically, the expression levels of *PLCβ* and *ARTISt* were downregulated, while the expression levels of *HB-EGF*, *EGFR*, *p38MAPK*, *PLA2*, *AC*, *cPLA*, and *COX-2* were upregulated. GO annotations indicated these genes are primarily involved in intracellular signal transduction and prostaglandin-endoperoxide synthase activity. Furthermore, the genes *ptgs2b*, *ptgs2a*, *ptges* related to prostaglandin E synthesis and PGE receptor *ptger1a* were also significantly upregulated (*P* < 0.01) (Table 2). The cAMP and cGMP-PKG signaling pathways are involved in the process of *ORs* binding with ligands. The genes *prkg1b*, *rgs2*, and *ncalda* which play an important role in the olfactory transduction, were upregulated after PGF_2α_ simulation (Supplementary Figure S4). To assess the reliability of sequencing and subsequent data analysis, 7 DGEs were randomly selected for validation through qPCR experiments. The qPCR verification results were in accordance with the RNA-Seq data, thus confirming the accuracy and dependability of RNA-Seq measurements (Figure 5D).

**TABLE 2:** Genes related to olfactory signal transduction after PGF_2α_ simulation.

Gene	Gene description	F vs. D	Log2FC
*slc8a1a*	solute carrier family 8-member 1a	down	−1.35
*prkg1b*	protein kinase cGMP-dependent 1b	up	2.63
*rgs2*	regulator of G protein signaling 2	up	2.05
*Ncalda*	neurocalcin delta a	up	1.08
*ptgs2a*	prostaglandin-endoperoxide synthase 2a	up	1.29
*ptgs2b*	prostaglandin-endoperoxide synthase 2b	up	2.76
*Ptges*	prostaglandin E synthase	up	2.34
*ptger1a*	prostaglandin E receptor 1a (subtype EP1)	up	1.57

**FIGURE 6:**
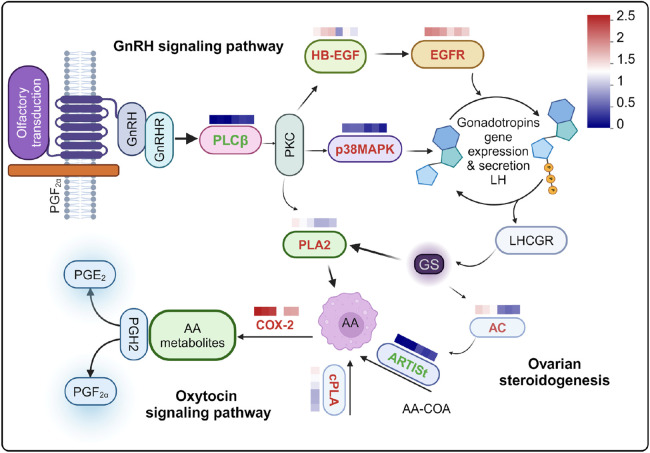
Relationships among reproductive-related pathways were examined following stimulation with PGF_2α_. Differentially expressed genes (DEGs) are indicated in bold font, with upregulated genes highlighted in red and downregulated genes highlighted in green. The heat map values represent log10 transformed absolute abundance. AA: Arachidonic acid. The heat map provides a comparison between PGF_2α_ and DMSO.

The olfactory epithelium cells exposed to PGF_2α_, DEGs was significantly enriched in ovarian steroidogenesis, GnRH signaling pathway, and oxytocin signaling pathway, all of which are highly interconnected. Consequently, we linked these pathways primarily through DEGs (Figure 6). We supposed that this pathway linkage elucidates the increased expression of PGE_2_ synthase and PGE receptors.

## 4 Discussion

Culturing mammalian OSNs *in vitro* poses an ongoing challenge in neurobiological research. Previous studies suggest that OSNs have limited proliferation and differentiation capacity *in vitro* (Kicic et al., 2006; Martinovich et al., 2017). The prevalence of immature OSNs in culture is believed to impair their odor detection capabilities, resulting in limited research on the cultivation of OSNs *in vitro*. However, recent findings by Huang et al. (2022) have revealed the previously unrecognized potential of immature OSNs to perceive odors, leading many researchers to reassess the value of cultivating OSNs *in vitro* (Huang et al., 2022). The processing capability of immature OSNs at high odor concentrations is crucial for odor-guided behavior essential for survival, particularly when olfactory function is compromised (Huang et al., 2022). Additionally, single-cell RNA sequencing results indicate that 55% of human OSNs are immature (Durante et al., 2020), highlighting the significance of immature OSNs in the olfactory system and emphasizing increased research attention and substantial progress made towards understanding mammalian olfaction. Research conducted in mammalian systems has revealed a direct correlation between the olfactory sensitivity of animals and the quantity of functional *ORs* they possess (Niimura and Nei, 2006). Additionally, there is a growing interest in studying the expression and function of *ORs* in other tissues (Wu et al., 2024). However, the research on OSNs in fish species remains relatively scarce compared to mammals. To enhance our understanding of fish olfaction recognition mechanisms through improved OSNs cultivation techniques, we selected the economically significant fish species *M. amblycephala* for the *in vitro* cultivation of olfactory epithelial cells.

In cell culture, explant and enzyme digestion are two commonly used techniques. The explant technique allows for continuous extraction of new cells from each explant, thereby significantly reducing harm to animal models. When utilizing the explant technique to cultivate olfactory epithelial cells of *M. amblycephala*, some neurons migrated out from the explants, exhibiting a morphology consistent with OSNs cultivated from mammal embryos (Micholt et al., 2012; Martinovich et al., 2017). However, the small number of cells derived from the explants resulted in a lack of cellular contact. Additionally, this phenomenon lacks consistency, as certain explants do not possess the capacity to migrate cells outward. Furthermore, through repeated experiments, we have confirmed that the olfactory epithelial explants of *M. amblycephala* do not exhibit neuronal migration. We posit that these differences in characteristics during *in vitro* culture stem from variances in tissue fragments originating from distinct regions of the olfactory epithelium. Due to differences in tissue and cellular specificity, it is speculated that culturing *M. amblycephala* OSNs using explants may not be suitable. We observed that the explant method exhibited a significantly higher contamination rate. This is likely due to the direct exposure of fish olfactory epithelial tissue to the aquatic environment, which presents a considerable challenge for explant culture.

Trypsinization exerts detrimental proteolytic effects, including the disruption of membrane protein integrity, alteration of cytoplasmic composition, and potential induction of cell death (Lordon et al., 2024). In mammals, collagenase I is primarily employed for dissociating epithelial cells (Lin et al., 2016), while collagenase IV is used for dissociating neurons. In the present study, we utilized a combination of collagenase I, collagenase IV, and trypsin-EDTA (0.25%) for the dissociation of olfactory epithelium cells (Lan et al., 2022). Prior to dissociation using collagenase, we excised the olfactory mucosa to minimize cell contamination (Randell et al., 2001). Following dissociation, olfactory epithelium cells were cultured in a growth medium containing 20% FBS for 24–36 h. The high concentration of FBS enhances cell adhesion (Fan and Karino, 2008). After adhesion, changing the neuronal culture medium facilitates neuronal growth. Upon observation, we discovered that olfactory epithelium cells obtained through collagenase digestion exhibit robust stability, accelerated growth, and distinctive neuronal morphological characteristics. SEM also revealed cells exhibiting the morphology of olfactory neurospheres (Li et al., 2018). In the olfactory epithelium tissue, sensory neurons constitute approximately 80% of the cell population (Bettina et al., 1999). NEUN is an antibody used as a maker for the identification of neuronal cells (Jiang et al., 2024). Most labeled cells are identified as neurons by this antibody staining method, while unlabeled cells are recognized as non-neuronal. Non-neuronal cells play a crucial role in promoting the growth and differentiation of OSNs (Dietz et al., 2023). OSNs express *ORs,* enabling them to recognize odor molecules (Buck and Axel, 1991; Niimura et al., 2014). FISH and RT-PCR detected the native expression of *ORs* in cultured cells derived from fish olfactory epithelial tissue, indicating that the cultured olfactory epithelial cells retain the ability to recognize odors.

Compared to DMSO, exposure to the amino acid mixture and PGF_2α_ significantly augmented the population of pERK-labeled olfactory epithelial cells. We propose that a subset of these labeled cells is stimulated by odor molecules, akin to the observations made in piscine olfactory organs (Wakisaka et al., 2017). Additionally, the RNA-seq results revealed the upregulation of the expression levels of *prkg1b*, *slc8a1a, ncalda* and *rgs2*, which play crucial roles in the olfactory transduction (Supplementary Figure S4). Previous studies have indicated that upon recognition of odor molecules by *ORs*, three key signal transduction pathways, namely, cAMP (Sands and Palmer, 2008), IP3(Fadool and Ache, 1992), and cGMP (Meyer et al., 2000), are activated. These activated specific signaling pathways reflect the characteristics of OSNs. In zebrafish, stimulation with PGF_2α_ has been observed to activate ciliated OSNs (Yabuki et al., 2016). Following activation, the G protein α subunit Golf converts intracellular ATP to cyclic adenosine monophosphate (cAMP) (Bakalyar and Reed, 1990). Elevated levels of intracellular cAMP then function as a secondary messenger, which triggers neural impulse generation within these cells (Restrepo et al., 1996; Sands and Palmer, 2008). However, some ciliated OSNs lack functional components required for the cAMP signaling pathway like Golf. In such cases, the cGMP signaling pathway serves as a crucial second messenger (Ma, 2007). Both pathways exhibit significant enrichment in the present study, leading us to speculate that PGF_2α_ activates the cultured OSNs.

PGF_2α_ acts as a sex pheromone (Lim and Sorensen, 2011; Sorensen et al., 2018), exerting influence on courtship behaviors in male zebrafish through the olfactory system (Yabuki et al., 2016). After exposure to PGF_2α_, there is an increase in PGE_2_ synthase and PGE receptor expression upon exposure to PGF_2α_. Recent findings indicate that PGE_2_ plays a role in synchronizing lunar-regulated beach-spawning behavior in grass puffers (Chen et al., 2022).

In summary, we employed collagenase digestion effectively to culture fish olfactory epithelial cells, and their morphological characteristics were validated using SEM. Following neuronal cell labeling with NEUN antibody, FISH and RT-PCR assays confirmed the expression of *ORs*. Although olfactory responsiveness was assessed within a relatively short time frame in cultured olfactory epithelial cells, this methodology represents a notable advancement in teleost olfactory research. It offers an alternative strategy to mitigate stress responses associated with *in vivo* fish experiments, establishes a novel platform for high-throughput receptor screening, and serves as a crucial foundation for conducting single-cell patch-clamp electrophysiology to study olfactory function *in vitro*. Future studies will focus on establishing continuous cell lines of fish OSNs to further enhance our understanding of their importance in survival and reproduction.

## Data Availability

The data presented in the study are deposited in the NCBI repository, accession number PRJNA1311481 at: https://dataview.ncbi.nlm.nih.gov/object/PRJNA1311481?reviewer=skf6gieokua7h11o64ukc9ifhr.

## References

[B1] Ahn J. H. Kim J. Hong S. P. Choi S. Y. Yang M. J. Ju Y. S. (2021). Nasal ciliated cells are primary targets for SARS-CoV-2 replication in the early stage of COVID-19. J. Clin. Invest 131, e148517. 10.1172/JCI148517 34003804 PMC8245175

[B2] Andrew C. Itamar S. Howard C. Axel R. (1994). Allelic inactivation regulates olfactory receptor gene expression. Cell 78 (5), 823–834. 10.1016/s0092-8674(94)90562-2 8087849

[B3] Bakalyar H. A. Reed R. R. (1990). Identification of a specialized adenylyl cyclase that may mediate odorant detection. Science. 250 (4986), 1403–1406. 10.1126/science.2255909 2255909

[B4] Bazáes A. Olivares J. Schmachtenberg O. (2013). Properties, projections, and tuning of teleost olfactory receptor neurons. J. Chem. Ecol. 39 (4), 451–464. 10.1007/s10886-013-0268-1 23468224

[B5] Bettina M. Junzo H. Takaaki S. Buck L. B. (1999). Combinatorial receptor codes for odors. Cell 96 (5), 713–723. 10.1016/s0092-8674(00)80581-4 10089886

[B6] Braubach O. R. Fine A. Croll R. P. (2012). Distribution and functional organization of glomeruli in the olfactory bulbs of zebrafish (*Danio rerio*). J. Comp. Neurol. 520 (11), 2317–2339. 10.1002/cne.23075 22581687

[B7] Buck L. Axel R. (1991). A novel multigene family may encode odorant receptors: a molecular basis for odor recognition. Cell 65 (1), 175–187. 10.1016/0092-8674(91)90418-x 1840504

[B8] Chen J. Katada Y. Okimura K. Yamaguchi T. Guh Y.-J. Nakayama T. (2022). Prostaglandin E_2_ synchronizes lunar-regulated beach spawning in grass puffers. Curr. Biol. 32 (22), 4881–4889.e5. 10.1016/j.cub.2022.09.062 36306789

[B9] Cong X. Zheng Q. Ren W. Chéron J.-B. Fiorucci S. Wen T. (2019). Zebrafish olfactory receptors *ORAs* differentially detect bile acids and bile salts. J. Biol. Chem. 294 (17), 6762–6771. 10.1074/jbc.RA118.006483 30833327 PMC6497948

[B10] Cui X. Chen L. Tao B. Zhang X. Song Y. Chen J. (2025). Olfactory GnRH3 crypt sensory neurons transduce sex pheromone signals to induce male courtship behavior in zebrafish. Sci. China Life Sci. 68, 2191–2205. 10.1007/s11427-025-2917-5 40347216

[B11] Dietz A. Senf K. Karius J. Stumm R. Neuhaus E. M. (2023). Glia cells control olfactory neurogenesis by fine-tuning CXCL12. Cells 12 (17), 2164. 10.3390/cells12172164 37681896 PMC10486585

[B12] Durante M. A. Kurtenbach S. Sargi Z. B. Harbour J. W. Choi R. Kurtenbach S. (2020). Single-cell analysis of olfactory neurogenesis and differentiation in adult humans. Nat. Neurosci. 23 (3), 323–326. 10.1038/s41593-020-0587-9 32066986 PMC7065961

[B13] Fadool D. A. Ache B. W. (1992). Plasma membrane inositol 1,4,5-Trisphosphate-Activated channels mediate signal transduction in lobster olfactory receptor neurons. Neuron 9 (5), 907–918. 10.1016/0896-6273(92)90243-7 1384577 PMC2843424

[B14] Fan L. Karino T. (2008). Effect of serum concentration on adhesion of monocytic THP-1 cells onto cultured EC monolayer and EC-SMC co-culture. J. Zhejiang Univ. Sci. B 9 (8), 623–629. 10.1631/jzus.B0820046 18763312 PMC2491692

[B15] Firestein S. (2001). How the olfactory system makes sense of scents. Nature 413, 211–218. 10.1038/35093026 11557990

[B16] Gao K. Gao F. Li J. He C. Liu M. Zhu Q. (2021). Biomimetic integrated olfactory sensory and olfactory bulb systems *in vitro* based on a chip. Biosens. Bioelectron. 171, 112739. 10.1016/j.bios.2020.112739 33096431

[B17] Hao S. Ning K. Kuz C. A. Vorhies K. Yan Z. Qiu J. (2020). Long-term modeling of SARS-CoV-2 infection of *in vitro* cultured polarized human airway epithelium. mBio 11 (6), e02852–20. 10.1128/mBio.02852-20 33158999 PMC7649230

[B18] Hou Y. J. Okuda K. Edwards C. E. Martinez D. R. Asakura T. Dinnon K. H. (2020). SARS-CoV-2 reverse genetics reveals a variable infection gradient in the respiratory tract. Cell 182 (2), 429–446. 10.1016/j.cell.2020.05.042 32526206 PMC7250779

[B19] Huang J. S. Kunkhyen T. Rangel A. N. Brechbill T. R. Gregory J. D. Winson-Bushby E. D. (2022). Immature olfactory sensory neurons provide behaviourally relevant sensory input to the olfactory bulb. Nat. Commun. 13 (1), 6194. 10.1038/s41467-022-33967-6 36261441 PMC9582225

[B20] Hughes G. M. Boston E. S. M. Finarelli J. A. Murphy W. J. Higgins D. G. Teeling E. C. (2018). The birth and death of olfactory receptor gene families in mammalian niche adaptation. Mol. Biol. Evol. 35 (6), 1390–1406. 10.1093/molbev/msy028 29562344 PMC5967467

[B21] Jiang H.-C. Park S. J. Wang I.-H. Bear D. M. Nowlan A. Greer P. L. (2024). CD20/MS4A1 is a mammalian olfactory receptor expressed in a subset of olfactory sensory neurons that mediates innate avoidance of predators. Nat. Commun. 15 (1), 3360. 10.1038/s41467-024-47698-3 38637611 PMC11026480

[B22] Kicic A. Sutanto E. N. Stevens P. T. Knight D. A. Stick S. M. (2006). Intrinsic biochemical and functional differences in bronchial epithelial cells of children with asthma. Am. J. Respir. Crit. Care Med. 174 (10), 1110–1118. 10.1164/rccm.200603-392OC 16908868

[B23] Lacroix M. C. Badonnel K. Meunier N. Tan F. Poupon S. L. Durieux D. (2008). Expression of insulin system in the olfactory epithelium: first approaches to its role and regulation. J. Neuroendocrinol. 20 (10), 1176–1190. 10.1111/j.1365-2826.2008.01777.x 18752648

[B24] Lan Y.-X. Yang P. Zeng Z. Yadav N. Zhang L.-J. Wang L.-B. (2022). Gene and protein expression profiles of olfactory ensheathing cells from olfactory bulb versus olfactory mucosa. Neural Regen. Res. 17 (2), 440–449. 10.4103/1673-5374.317986 34269221 PMC8463967

[B25] Li S. T. Young T. H. Wang C. T. Huang T. W. (2018). Chitosan films promote formation of olfactory neurospheres and differentiation of olfactory receptor neurons. Rhinology 56 (4), 336–342. 10.4193/Rhin17.155 30052693

[B26] Lim H. Sorensen P. W. (2011). Polar metabolites synergize the activity of prostaglandin F_2α_ in a species-specific hormonal sex pheromone released by ovulated common carp. J. Chem. Ecol. 37 (7), 695–704. 10.1007/s10886-011-9976-6 21647722

[B27] Lin J. Yang L. Silva H. M. Trzeciak A. Choi Y. Schwab S. R. (2016). Increased generation of Foxp3^+^ regulatory T cells by manipulating antigen presentation in the thymus. Nat. Commun. 7, 10562. 10.1038/ncomms10562 26923114 PMC4773449

[B28] Liu H. Chen C. Lv M. Liu N. Hu Y. Zhang H. (2021). A chromosome-level assembly of blunt snout bream (*Megalobrama amblycephala*) genome reveals an expansion of olfactory receptor genes in freshwater fish. Mol. Biol. Evo 38 (10), 4238–4251. 10.1093/molbev/msab152 34003267 PMC8476165

[B29] Lordon B. Campion T. Gibot L. Gallot G. (2024). Impact of trypsin on cell cytoplasm during detachment of cells studied by terahertz sensing. Biophys. J. 123 (16), 2476–2483. 10.1016/j.bpj.2024.06.011 38877703 PMC11365101

[B30] Ma M. (2007). Encoding olfactory signals via multiple chemosensory systems. Crit. Rev. Biochem. Mol. 42 (6), 463–480. 10.1080/10409230701693359 18066954

[B31] Martinovich K. M. Iosifidis T. Buckley A. G. Looi K. Ling K.-M. Sutanto E. N. (2017). Conditionally reprogrammed primary airway epithelial cells maintain morphology, lineage and disease specific functional characteristics. Sci. Rep. 7 (1), 17971. 10.1038/s41598-017-17952-4 29269735 PMC5740081

[B32] Masuda M. Ihara S. Mori N. Koide T. Miyasaka N. Wakisaka N. (2024). Identification of olfactory alarm substances in zebrafish. Cur Biol. 34 (7), 1377–1389.e7. 10.1016/j.cub.2024.02.003 38423017

[B33] Meyer M. R. Angele A. Kremmer E. Kaupp U. B. Müller F. (2000). A cGMP-signaling pathway in a subset of olfactory sensory neurons. Proc. Natl. Acad. Sci. U.S.A. 97 (19), 10595–10600. 10.1073/pnas.97.19.10595 10984544 PMC27070

[B34] Micholt E. Jans D. Callewaert G. Bartic C. Lammertyn J. Nicolai B. (2012). Primary culture of embryonic rat olfactory receptor neurons. Vitro Cell Dev. Biol. 48 (10), 650–659. 10.1007/s11626-012-9560-6 23150136

[B35] Murrell W. Féron F. Wetzig A. Cameron N. Splatt K. Bellette B. (2005). Multipotent stem cells from adult olfactory mucosa. Dev. Dyn. 233 (2), 496–515. 10.1002/dvdy.20360 15782416

[B36] Niimura Y. Nei M. (2006). Evolutionary dynamics of olfactory and other chemosensory receptor genes in vertebrates. J. Hum. Genet. 51, 505–517. 10.1007/s10038-006-0391-8 16607462 PMC1850483

[B37] Niimura Y. Matsui A. Touhara K. (2014). Extreme expansion of the olfactory receptor gene repertoire in African elephants and evolutionary dynamics of orthologous gene groups in 13 placental mammals. Genome Res. 24 (9), 1485–1496. 10.1101/gr.169532.113 25053675 PMC4158756

[B38] Peterson J. Lin B. Barrios-Camacho C. M. Herrick D. B. Holbrook E. H. Jang W. (2019). Activating a reserve neural stem cell population *in vitro* enables engraftment and multipotency after transplantation. Stem Cell Rep. 12 (4), 680–695. 10.1016/j.stemcr.2019.02.014 30930245 PMC6450498

[B39] Policarpo M. Baldwin M. W. Casane D. Salzburger W. (2024). Diversity and evolution of the vertebrate chemoreceptor gene repertoire. Nat. Commun. 15 (1), 1421. 10.1038/s41467-024-45500-y 38360851 PMC10869828

[B40] Randell S. H. Walstad D. L. Schwab U. E. Yankaskas G. J. R. Yankaskas J. R. (2001). Isolation and culture of airway epithelial cells from chronically infected human lungs. Vitro Cell Dev. Biol. Anim. 37 (8), 480–489. 10.1290/1071-2690(2001)037<0480:iacoae>2.0.co;2 11669281

[B41] Ren W. Wang L. Zhang X. Feng X. Zhuang L. Jiang N. (2021). Expansion of murine and human olfactory epithelium/mucosa colonies and generation of mature olfactory sensory neurons under chemically defined conditions. Theranostics 11 (2), 684–699. 10.7150/thno.46750 33391499 PMC7738855

[B42] Restrepo D. Teeter J. H. Schild D. (1996). Second messenger signaling in olfactory transduction. J. Neurobiol. 30 (1), 37–48. 10.1002/(SICI)1097-4695(199605)30:1<37::AID-NEU4>3.0.CO;2-H 8727981

[B43] Sands W. A. Palmer T. M. (2008). Regulating gene transcription in response to cyclic AMP elevation. Cell Signal 20 (3), 460–466. 10.1016/j.cellsig.2007.10.005 17993258

[B44] Schmittgen T. D. Livak K. J. (2008). Analyzing real-time PCR data by the comparative CT method. Nat. Protoc. 3 (6), 1101–1108. 10.1038/nprot.2008.73 18546601

[B45] Schwarzenbacher K. Fleischer J. Breer H. (2005). Formation and maturation of olfactory cilia monitored by odorant receptor-specific antibodies. Histochem Cell Biol. 123 (4-5), 419–428. 10.1007/s00418-005-0790-5 15868179

[B46] Soelter J. Schumacher J. Spors H. Schmuker M. (2020). Computational exploration of molecular receptive fields in the olfactory bulb reveals a glomerulus-centric chemical map. Sci. Rep. 10 (1), 77. 10.1038/s41598-019-56863-4 31919393 PMC6952415

[B47] Sorensen P. W. Appelt C. Stacey N. E. Goetz F.Wm. Brash A. R. (2018). High levels of circulating prostaglandin F_2α_ associated with ovulation stimulate female sexual receptivity and spawning behavior in the goldfish (*Carassius auratus*). Gen. Comp. Endocrinol. 267, 128–136. 10.1016/j.ygcen.2018.06.014 29940184

[B48] Suzuki Y. Takeda M. (1993). Basal cells in the mouse olfactory epithelium during development: immunohistochemical and electron-microscopic studies. Brain Res. Dev. Brain Res. 73 (1), 107–113. 10.1016/0165-3806(93)90052-c 7685663

[B49] Wakisaka N. Miyasaka N. Koide T. Masuda M. Hiraki-Kajiyama T. Yoshihara Y. (2017). An adenosine receptor for olfaction in fish. Cur Biol. 27 (10), 1437–1447. 10.1016/j.cub.2017.04.014 28502661

[B50] Wu C.-T. Lidsky P. V. Xiao Y. Cheng R. Lee I. T. Nakayama T. (2023). SARS-CoV-2 replication in airway epithelia requires motile cilia and microvillar reprogramming. Cell 186 (1), 112–130.e20. 10.1016/j.cell.2022.11.030 36580912 PMC9715480

[B51] Wu C. Xu M. Dong J. Cui W. Yuan S. (2024). The structure and function of olfactory receptors. Trends Pharmacol. Sci. 45 (3), 268–280. 10.1016/j.tips.2024.01.004 38296675

[B52] Yabuki Y. Koide T. Miyasaka N. Wakisaka N. Masuda M. Ohkura M. (2016). Olfactory receptor for prostaglandin F_2α_ mediates male fish courtship behavior. Nat. Neurosci. 19 (7), 897–904. 10.1038/nn.4314 27239939

