# Supplementary material for: In vitro culture of olfactory epithelial cells from Megalobrama amblycephala and their response to amino acid mixtures and prostaglandin F2α
Source: Front Cell Dev Biol. 2025 Sep 11;13:1587151. doi: 10.3389/fcell.2025.1587151 (PMC12460313; doi:10.3389/fcell.2025.1587151)
Supplement: Supplementary file 1 [file DataSheet1.pdf]

Supplementary Materials

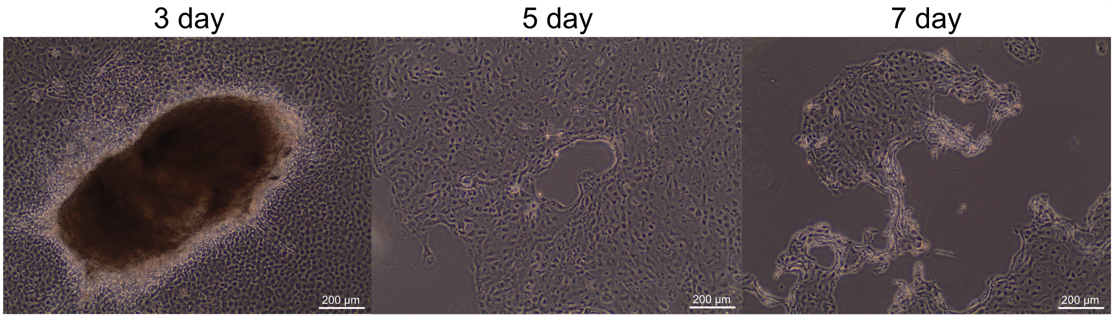

Figure S1. Depicts the explant sheathed by paving-stone like cells.

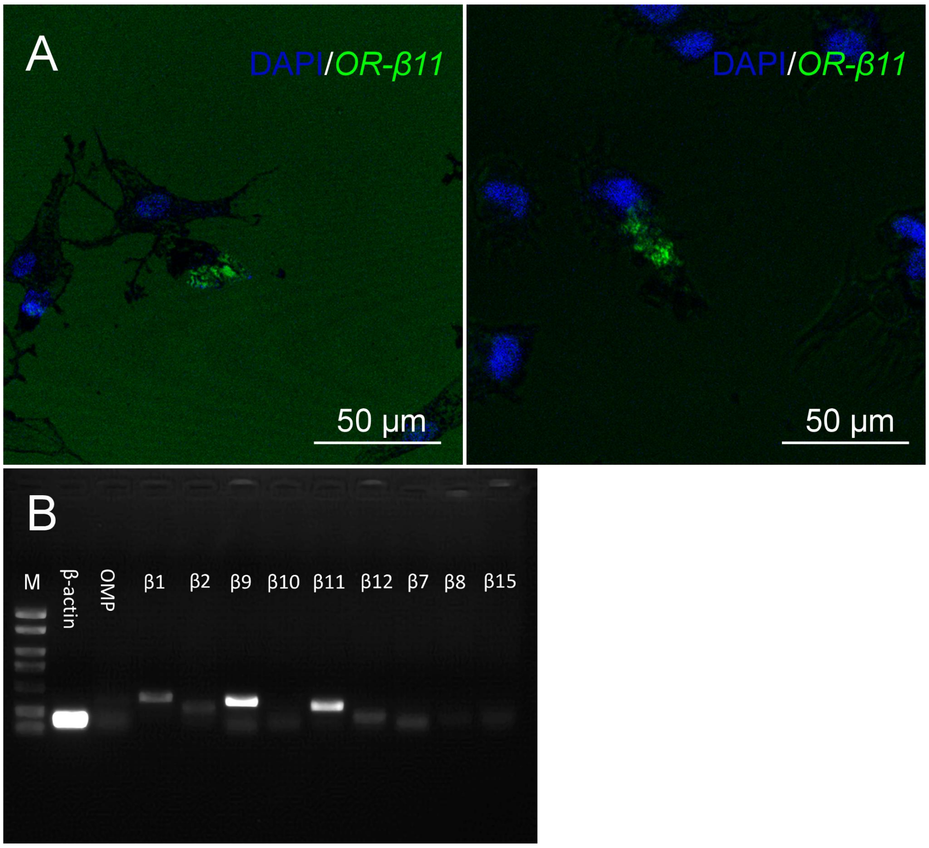

Figure S2. Detection of olfactory receptors (*ORs*) in an *in vitro* model of the olfactory epithelium in *M. amblycephala*. (A) Expression of *OR-β11* (*OR114-1*) was assessed in cells using fluorescence in situ hybridization (FISH). (B) The expression patterns of *OR-β* subtype in the cells were analyzed using RT-PCR.

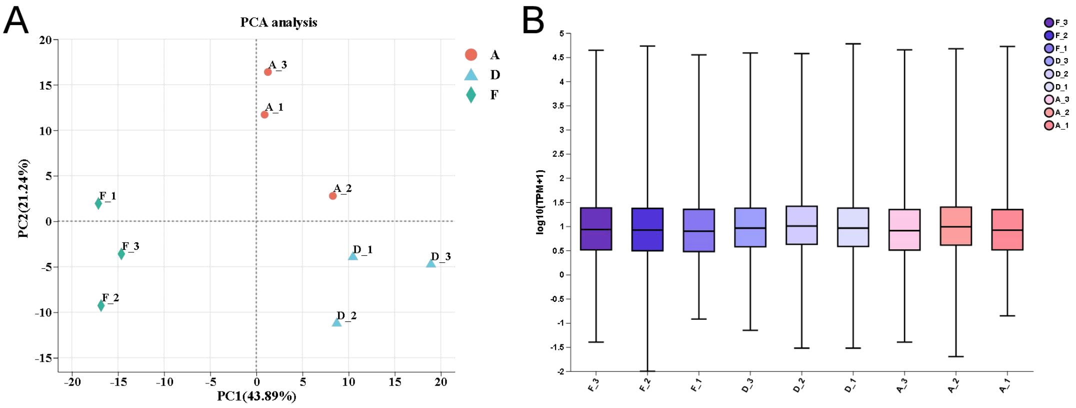

Figure S3. RNA-seq data. (A) Principal Component Analysis (PCA) plot depicting sample relationships. (B) Distribution of gene expression levels in each sample. The x-axis shows the sample names, and the y-axis shows log10 (TPM+1).

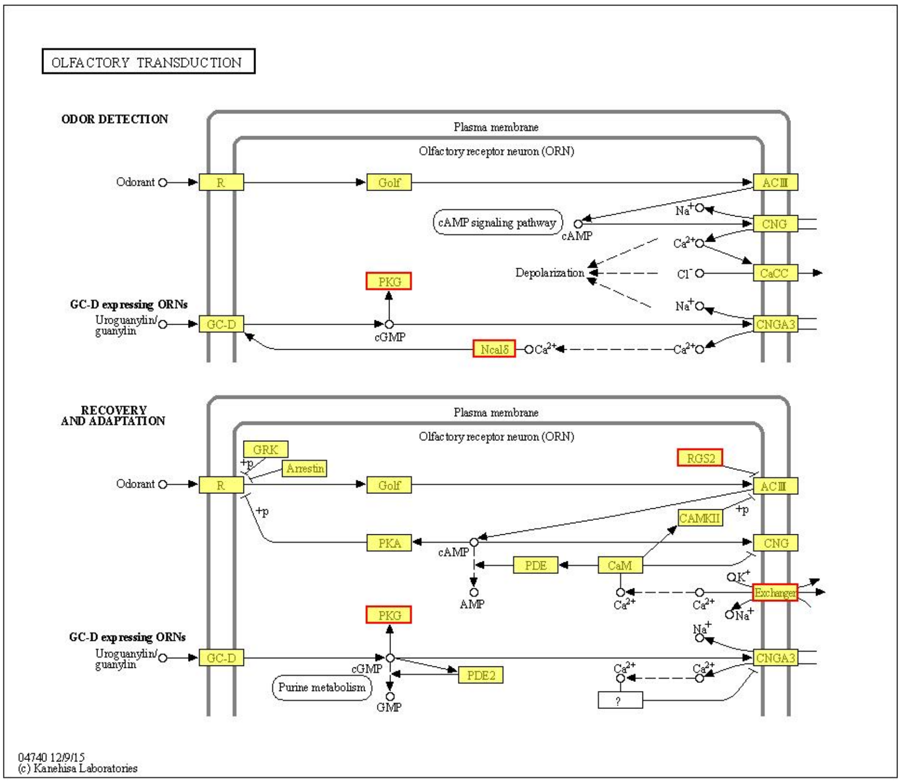

Figure S4. Olfactory transduction pathway diagram. Genes marked in red represent DEGs upregulated following $PGF_{2\alpha}$ stimulation.

Table S1. Composition of amino acid mixture

CAS	Amino acid	Chemical formula	Molecular weight	Proportion %	Structural formula
56-45-1	L-Serine	$C_3H_7NO_3$	105.09	55.33	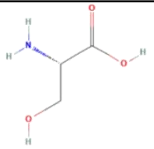
56-84-8	Aspartic acid	$C_4H_7NO_4$	133.103	19.54	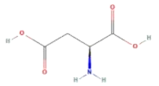
63-91-2	Phenylalanine	$C_9H_{11}NO_2$	165.19	12.51	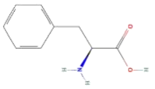
71-00-1	Histidine	$C_6H_9N_3O_2$	155	7.20	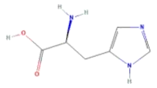
7004-03-7	Valine	$C_5H_{11}NO_2$	117.146	5.41	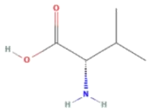

The selection and proportion of the amino acid mixture were based on the amino acid composition analysis of *Hydrilla verticillata* (unpublished data).

Table S2. FISH, qPCR and RT-PCR primers

Experimental method	Gene name	Primer Sequence (5'-3')
FISH	*OR-β11*	F: TTTTCATTCCTTTTCTC
		R: TAATACGACTCACTATAGGG TGATTTCCTTTGTTCTT
RT-PCR	*β-actin*	F: ACCCACACCGTGCCCATCTA
		R: CGGACAATTTCTCTTTCGGCTG
	OMP	F: CCCTGACATGCAGCTGACAG
		R: GCGCTTTTCCAGAAGAGACC
	OR-β1	F: GGGGAATGGAGGCCCTTTTT
		R: CAGTGCCATCCACAAAAGCA
	OR-β2	F: TTGTGGATGGCACTGGATCG
		R: TGCCATGTGCTCACAAAAACA
	OR-β9	F: GCTTTTGTGGATGGCACTGG
		R: AGTCTGCAGTTGGGATCAGA
	OR-β10	F: GGCTTTAGATCGCTTTGCGG
		R: CACAGGCCAGGCTTACAAGA

	OR-β11	F: CCGTAACTTGCTTTGTGTTGT
		R: GACTCACCAGAAGGAGAGCG
	OR-β12	F: TCCACCATCTTGCTTGGGAT
		R: CAACTAGGCCAACCATGGCA
	OR-β7	F: CCTGGTGGTCATTACGGTCTC
		R: CCCCACATGCTAACTGAACCA
	OR-β8	F: GTGGATGGCACTGGATCGTTA
		R: TTTCCAGCCAGAGAGACTGTG
	OR-β15	F: GAGCACATGGCATTGGTTCA
		R: AGGCCTTCACGTGAGCTTT
qPCR	*lamb3*	F: CGGATGGGAGTATGT
		R: GCGTTGAGGTTGTAG
	ptges	F: AGGACGCCGACAGAC
		R: CGACCCAGGAAGAAG
	s100a10a	F: CTGGAAAAGAGGGCA
		R: TCTATGGTGGTGGGA
	egfra	F: TCCTGGTCCCACTAT
		R: TTCGACACACGTTTT
	atp1a3b	F: GCTGCTTCTCCTACT
		R: GCATCTTCTCTCCTT
	anks1b	F: AACCCCCTCCTTTAC
		R: AACCACTGTCCCACG
	ptgs2b	F: CCAGTCTTATGTTCG
		R: CTTGTGTTGGCGTTC
